# Loneliness and Social Isolation among Transgender and Gender Diverse People

**DOI:** 10.3390/healthcare11101517

**Published:** 2023-05-22

**Authors:** André Hajek, Hans-Helmut König, Marco Blessmann, Katharina Grupp

**Affiliations:** 1 Department of Health Economics and Health Services Research, University Medical Center Hamburg-Eppendorf, Hamburg Center for Health Economics, 20246 Hamburg, Germany; 2 Division of Plastic, Reconstructive and Aesthetic Surgery, University Medical Center Hamburg-Eppendorf, 20246 Hamburg, Germany

**Keywords:** loneliness, social isolation, social exclusion, social embeddedness, transgender and gender diverse, sexual minority, transgender

## Abstract

Here, we report the prevalence of loneliness and social isolation and investigate the levels of loneliness and social isolation among transgender and gender diverse people using cross-sectional data from the HH-TPCHIGV study. Using the De Jong Gierveld tool, we assess loneliness, using the Bude and Lantermann tool, we assess perceived social isolation and using the Lubben Social Network Scale, we assess objective social isolation. The prevalence rate of loneliness was 83.3% (perceived social isolation: 77.7%; objective social isolation: 34.4%). Regressions revealed that favorable outcomes (i.e., lower loneliness levels, lower perceived social isolation, and lower objective social isolation) were consistently associated with higher school education. Beyond that, we identify an association between particularly poor health-related factors and higher loneliness and objective social isolation levels. We also report that unemployment was significantly associated with higher levels of perceived social isolation. In conclusion, we show high prevalence rates of loneliness and social isolation among transgender and gender diverse people. Additionally, important correlates (e.g., education, health-related factors, or unemployment) were identified. Such knowledge may provide help to address transgender and gender diverse people at risk for loneliness and social isolation.

## 1. Introduction

Transgender and gender diverse people identify with a different gender from their sex assigned at birth [1]. Trans feminine describes transgender people assigned a male sex at birth who identify as female and trans masculine describes transgender people assigned a female sex at birth who identify as men. For transgender and gender diverse people health inequities are multifactorial and include increased risks for systematic social and economic marginalization, stigma, and discrimination resulting in increased risk for various negative mental and physical health outcomes [2].

The minority stress theory provides a theory to acquire more information about the life experience of transgender and gender diverse people living in society [3,4]. The theory suggests that chronic internal and external stressors (e.g., internalized transphobia, perceived stigma, discrimination, rejection) of transgender and gender diverse people causes mental and physical health problems [5]. These mental and physical health problems include depression and loneliness [6].

Worldwide gender minorities become more visible. Transgender and gender diverse people are part of a socially marginalized group [7]. Because of chronic marginalization and isolation, individuals can feel lonely [8]. Even in the absence of social separation, the feeling of loneliness may result from an unfriendly environment that lacks affectionate care and help. Social isolation is associated with increased mortality and several illnesses, for example, cardiovascular and mental health diseases [9]. Objective social isolation is defined as having a low quantity and quality of contact with others. It can be measured using the number of persons in an individual’s social network [10]. Perceived social isolation refers to a feeling of not belonging to the society [11]. Loneliness is the feeling of discrepancy between actual and desired social relations [12]. Actual and perceived social isolation are both associated with increased risk for mortality and morbidity [13,14]. Thus, loneliness is associated with several negative health outcomes including coronary heart disease and stroke [15], dementia [16], poor mental health [9], and metabolic syndrome [17].

Studies on transgender and gender diverse people showed that loneliness was higher in transgender and gender diverse people living in Pakistan [8], Norway [18], the United States [19], and Australia [20] compared with cisgender peers. For example, Anderssen et al. [18] showed that 38–52% of binary transgender and gender diverse individuals reported often or very often either “lacking companionship,” “feeling left out” or “feeling isolated from others,” and that similar rates were observed for non-binary transgender people (38–48%), while the corresponding rates for cisgender males and females were 15–21% and 17–24%. Allen et al. [19] demonstrated among more than 4000 transgender, nonbinary, and gender-diverse adolescents in the United States that these people had higher scores for loneliness compared with cisgender peers. Moreover, Eres et al. [20] showed higher levels of loneliness, depression, and social anxiety in lesbian, gay, bisexual, transgender, queer, intersex, asexual, and other sexual orientation and gender identity diverse people (LGBTQIA+) in Australia than the non-LGBTQIA+ comparison group.

To our best knowledge, there is a dearth of knowledge about the levels of loneliness and isolation among transgender and gender diverse people in Germany.

## 2. Materials and Methods

### 2.1. Sample

Cross-sectional data were taken from the HH-TPCHIGV study—which is a joint project of the “Department of Health Economics and Health Services Research” and the “Division for Plastic, Reconstructive, and Esthetic Surgery”. They are both located at the University Medical Center Hamburg-Eppendorf (UKE), Hamburg (Germany). With regard to inclusion: transgender and gender diverse individuals who have joined together in self-help groups on Whatsapp and Facebook, among other places, in order to exchange information about trans-specific operations at the UKE in the Department of Plastic, Reconstructive and Aesthetic Surgery were surveyed. 

No specific exclusion criteria (e.g., with regard to age) were applied. Data collection took place from April to October 2022. We used the online survey tool “Limesurvey” for programming, hosting, and performing the survey.

Informed consent was obtained from all participants included in the study. Approval for the study was provided by the Local Psychological Ethics Committee of the Center for Psychosocial Medicine of the University Medical Center Hamburg-Eppendorf (number: LPEK-0480).

### 2.2. Dependent Variables

To measure loneliness, the De Jong Gierveld Loneliness tool was used [21]. It consists of six items. Thereof, three items were recoded, whereas three items were not recoded. By averaging the items, a final score was computed. This final score ranged from 0 to 6, with higher values reflecting higher loneliness. Scores of 0 to 1 were used to categorize individuals as ‘not lonely’. In contrast, higher scores were used to categorize individuals as ‘lonely’—as suggested in prior research [22]. In this study, Cronbach’s alpha was 0.84 (McDonald’s omega was also 0.84). This tool has favorable psychometric characteristics [21].

The Bude and Lantermann tool was used to measure perceived social isolation [11]. It has four items. The score was computed by averaging the items. This score ranged from 0 to 6, with higher values corresponding to higher perceived social isolation. Analogously, we also categorized individuals as ‘not socially isolated (perceived)’ if scores ranged from 0 to 1 [23]. In contrast, they were categorized as ‘socially isolated (perceived)’ if their scores were greater. In this study, Cronbach’s alpha was 0.89 and McDonald’s omega equaled 0.89.

To quantify objective social isolation, we used the 6-item version of the Lubben Social Network Scale (6-item version) [24]. Based on the six items, a sum score was created. It ranges from 0 to 30 (with higher values reflecting lower objective social isolation). In line with former recommendations [24], a score below 12 was used to classify individuals as ‘socially isolated (objective)’. In contrast, higher values were used to classify individuals as ‘not socially isolated (objective)’. In this study, Cronbach’s alpha was 0.80 and McDonald’s omega equaled 0.77). Favorable psychometric characteristics have been documented for this tool [24].

### 2.3. Determinants

Inspired by former research in this field [25,26,27], sociodemographic, lifestyle-related as well as health correlates were covered (in our regressions): age, marital situation (single; widowed; divorced; separately: married or in partnership; living together: married/in partnership), school level (without general school leaving certificate; intermediate school leaving certificate; secondary school diploma; currently in school; polytechnic secondary school; general or subject-specific university entrance qualification), employment situation (marginally employed (450-euro job or mini-job); retired; in retraining; unemployed; vocational training/apprenticeship; part-time employed; full-time employed; other), religious affiliation (non-denominational; Buddhism; Islam; Christianity; Other), having a migration background (no; yes), gender reassignment surgery (no; yes), sports activities (from: 1 (“no sports activity”) to 5 (“more than 4 h a week”), self-rated health (1 = very bad to 5 = very good, single-item question), and at least one chronic condition (no; yes).

### 2.4. Statistical Analysis

In the first step, the prevalence rate of loneliness, perceived social isolation, and objective social isolation were displayed (for certain important groups). Thereafter, multiple linear regression analysis was used to examine the determinants of the levels of loneliness, perceived social isolation, and objective social isolation.

We used a tool which was created by Shaw [28] to compute McDonald’s omega. A full-information maximum likelihood (FIML) approach was used to tackle missing data [29]. Statistical significance was defined as a *p*-value of 0.05 or smaller. Stata 16.1 (Stata Corp., College Station, TX, USA) was used to conduct statistical analyses.

## 3. Results

### 3.1. Sample Characteristics, Prevalence Rates, and Pairwise Correlations for the Outcome Measures

The mean age was 30.4 years (19 to 63 years, SD: 9.6 years) in this sample. About half of the individuals included in the sample had at least one chronic condition. More than half of the individuals did not have a general or subject-specific university entrance qualification. Additional details regarding the sample characteristics are shown in Table 1.

More generally, the prevalence rates are shown in Table 1. We also reported prevalence rates by important groups. The prevalence rate of loneliness was 83.3% (perceived social isolation: 77.7%; objective social isolation: 34.4%). The prevalence rates oscillate between the groups examined. For instance, the prevalence rate of objective social isolation was 13.0% among individuals without chronic diseases, whereas it was 56.3% among individuals with at least one chronic disease. Please see Table 1 for further details.

The Pearson correlation between loneliness and perceived social isolation was r = 0.69, *p* < 0.001. Moreover, the Pearson correlation between loneliness and objective social isolation was r = −0.50, *p* < 0.001 (worth repeating that the negative sign just reflects that higher scores on the LSNS-6 tool correspond to lower objective social isolation). The Pearson correlation between perceived social isolation and objective social isolation was r = −0.37, *p* < 0.001.

### 3.2. Regression Analysis

In Table 2, the results of multiple linear regressions are given (outcomes: loneliness, perceived social isolation, and objective social isolation). Unstandardized beta-coefficients are shown in Table 2 (and robust standard errors are in parentheses). R^2^ varied from 0.30 (with objective social isolation as an outcome measure) to 0.35 (with perceived social isolation as an outcome measure). Regressions revealed that favorable outcomes (i.e., lower loneliness levels (β = −0.47, *p* < 0.01), lower perceived social isolation (β = −0.45, *p* < 0.05) and lower objective social isolation (β = 2.67, *p* < 0.05)) were only consistently associated with higher school education.

Beyond that, higher loneliness levels were significantly associated with being unmarried (β = −0.30, *p* < 0.05), already having a gender reassignment surgery (β = 0.30, *p* < 0.05), more than 4 h a week of sports activities (compared to no sports activity, β = 0.36, *p* < 0.05), and poor self-rated health (β = −0.18, *p* < 0.05). Moreover, higher perceived social isolation levels were associated with being unemployed (e.g., compared to full-time employed, β = −0.75, *p* < 0.001), and 1–2 h a week of sports activities (compared to no sports activity, β = 0.45, *p* < 0.05). Additionally, higher objective social isolation levels were also associated with having at least one chronic condition (compared to the absence of chronic conditions, β = −3.73, *p* < 0.01).

## 4. Discussion

Using data from a hospital-based sample, our aim was to examine the prevalence of loneliness and social isolation and to investigate the levels of loneliness and social isolation among transgender and gender diverse people in Germany. Here, we identify high to very high prevalence rates of loneliness and social isolation among transgender and gender diverse people. Moreover, higher levels of loneliness and objective social isolation were associated with poor health-related factors and lower school education. Our study adds to our current knowledge by analyzing the prevalence of loneliness and social isolation in a cohort of transgender people in Germany.

Our result of the high prevalence of loneliness and social isolation in transgender and gender diverse people in Germany is consistent with the literature currently demonstrating that levels of loneliness were higher in transgender and gender diverse people compared with cisgender people in several other countries [8,18,19]. For example, Batool et al. [8] described a mean of 47.6 among 200 transgender and gender diverse people and a mean of 39.7 among 100 cisgender people in Pakistan using the UCLA Loneliness Scale. Anderssen et al. [18] showed that the level of loneliness (The Three-Item Loneliness Scale) was significantly higher in transgender and gender diverse people as compared to cisgender people in Norway. Rutter et al. [30] performed a cross-sectional analysis including 4592 American adolescents and showed that transgender and gender diverse adolescents had higher rates of loneliness than the female and male adolescents in the sample. Moreover, Allen et al. [19] surveyed a US nationally representative sample of 4575 adolescents (aged 13–18 years) and showed that transgender and gender diverse and nonbinary youth had higher scores for loneliness as compared to cisgender people. Our result indicates a vulnerable gender minority population group in need of special attention in Germany as well as in other countries.

The prevalence rates should also be seen in the light of a potential positive marginality [31]. For example, adopting a marginalized identity can improve the life of transgender and gender diverse people by increasing their self-confidence, positive body image, and sense of serenity. Moreover, belonging to a minority group can facilitate the development of meaningful relationships with others [31].

Our study aimed to identify determinants of loneliness and social isolation among transgender and gender diverse people. In general, the feeling of loneliness often leads to coping behaviors to reduce these feelings. Coping behaviors attempt to manage stressful situations and include adaptive and maladaptive strategies. Adaptive strategies include for example active problem solving, and seeking help and support. Using these strategies results in enhanced psychological health and self-esteem [32]. In comparison to adaptive strategies, maladaptive strategies such as denial or avoidance, substance abuse, aggression, and escape often result in enhanced levels of distress for individuals [32].

In our study, levels of loneliness and social isolation were reduced in individuals with higher school education. It can be speculated that higher school education may serve as a protective factor for isolation and loneliness. Previously, authors have shown that people with higher school education have better-coping skills to deal with negative experiences compared to individuals with lower school education [33]. Uncertainty exists, however, about the specific elements of education that influence loneliness and social isolation. Another way to explain this link may be that higher loneliness is associated with higher levels of discrimination from associates who belong to educationally disadvantaged classes. More precisely, if one has a lower level of education, one may associate with people who also have a lower level of education. If those people discriminate against transgender and gender diverse people, one may face additional discrimination. However, additional research is required to test these pathways.

Interestingly, our study also showed that transgender and gender diverse people, who are unmarried were characterized by higher levels of loneliness, and those who were unemployed were characterized by higher levels of perceived social isolation. Lack of support (in different areas of life such as family situations or labor force participation) is associated with factors such as low general self-esteem or low mental health [33,34].

Additionally, our data indicate that transgender and gender diverse people, who have undergone gender reassignment surgery feel lonelier. To our knowledge, this is the first study analyzing the levels of loneliness and social isolation in operated transgender and gender diverse people. Previously, van de Grift et al. [35] suggested that multiple stressors occur throughout an individual’s gender-affirming treatment resulting in a significant number of relapses or persistence of psychiatric problems of people. Such factors may contribute to higher loneliness levels. However, future research is required to examine this association in further detail.

Moreover, we found a significant association between having at least a chronic disease and higher levels of objective social isolation. Several factors and problems are associated with chronic illnesses and might cause higher objective isolation levels. For example, people with chronic illnesses usually require more care [35]. The higher demand for care of individuals with chronic conditions may also reflect the fact that their social relationships are more frequently restricted to professionals (e.g., outpatient care services)—particularly in times of the COVID-19 pandemic [36]. This could contribute to dissatisfaction (in terms of social needs) and thus intensify feelings of social isolation [36].

Moreover, poor self-rated health was associated with higher loneliness levels in our study. A potential explanation for this association may be as follows: Prior research has shown that functional impairment is a key factor to explain self-rated health [37]. By restricting the opportunities for social activities due to such functional impairment, feelings of loneliness may develop in the affected individuals [38].

Moreover, gender minority people/individuals experience many barriers to accessing resources including healthcare and education, and often face discrimination and negative experiences when seeking support. According to the minority stress model [39], the behavior that results from negative experiences places minority gender and gender diverse people at further risk, perpetuating a cycle of inadequate care, deteriorating health, and a diminished sense of well-being [40]. Several stressors have a negative impact on the ability to cope and consequently, their health-averse behaviors increase leading to further victimization and discrimination [41]. Thus, transgender and gender diverse people face isolation resulting in feelings of isolation with increased risk for physical and psychical health. In the case of social isolation and feeling of loneliness, adaptive coping strategies and resilience can help to improve the situation.

It should be noted that Bailey emphasized that the minority stress theory needs reconsideration [42]. It has several limitations (e.g., this model relied solely on self-report data to measure stigmatization) [42]. Alternative models that acknowledge temperament could deserve more attention [43].

When assessing our current findings, it is important to keep in mind several strengths and limitations. Widely used tools with favorable psychometric properties were used to quantify loneliness, perceived social isolation, and objective social isolation. Moreover, a wide array of correlates was explored and FIML was used to address missing data. It should be acknowledged that this study has a cross-sectional design. This makes it difficult to clarify the directionality of the factors associated with loneliness, perceived social isolation, and objective social isolation. For example, the outcomes used may also contribute to chronic conditions [44]. Future longitudinal studies are required to confirm our present findings. Additionally, data were taken from a hospital-based sample - which can have consequences for the generalizability. Furthermore, also because the sample size of our current study was quite low, large, nationally representative studies are needed to verify our results.

## 5. Conclusions and Future Research

In our study, high prevalence rates of loneliness and social isolation were shown among transgender and gender diverse people. These high levels of loneliness and objective social isolation were associated with poor health-related factors and lower school education. Such knowledge can assist to address transgender and gender diverse people with a high likelihood of loneliness and social isolation.

With regard to future research, qualitative studies are of particular interest to better understand the pressing topic of loneliness and social isolation among transgender and gender diverse people.

## Figures and Tables

**Table 1:** Prevalence: loneliness, perceived social isolation, and objective social isolation among several subgroups.

	*n*	Loneliness	*p*-Value	Perceived Social Isolation	*p*-Value	Objective Social Isolation	*p*-Value
Total sample	*n* = 96	83.3%		77.7%		34.4%	
Age bracket			0.67		0.28		0.24
18 to 29 years	*n* = 53	84.9%		83.0%		30.2%	
30 years and older	*n* = 38	81.6%		73.7%		42.1%	
Family situation			0.08		0.69		0.92
Living separately: married or in partnership; divorced; single; widowed	*n* = 49	89.8%		77.6%		34.7%	
Married or in partnership	*n* = 42	76.2%		81.0%		35.7%	
School education			<0.01		0.14		<0.001
Absence of general or subject-specific university entrance qualification	*n* = 52	92.3%		84.6%		50.0%	
Presence of general or subject-specific university entrance qualification	*n* = 39	71.8%		71.8%		15.4%	
Labor force participation			0.44		0.08		0.67
Unemployed	*n* = 16	93.8%		93.8%		37.5%	
Full-time employed	*n* = 34	79.4%		67.6%		29.4%	
Other	*n* = 41	82.9%		82.9%		39.0%	
Migration background			0.87		0.31		0.93
No	*n* = 80	83.8%		77.5%		35.0%	
Yes	*n* = 11	81.8%		90.9%		36.4%	
Having a religious affiliation			0.67		0.58		0.47
Non-denominational	*n* = 53	84.9%		81.1%		32.1%	
Having a religious affiliation	*n* = 38	81.6%		76.3%		39.5%	
Already having a gender reassignment surgery			0.91		0.78		0.91
No	*n* = 50	83.3%		79.2%		35.4%	
Yes	*n* = 38	84.2%		81.6%		34.2%	
Chronic diseases			0.27		0.08		<0.001
Absence of at least one chronic disease	*n* = 52	79.2%		70.2%		13.0%	
Presence of at least one chronic disease	*n* = 50	87.5%		85.1%		55.3%	

Notes: With regard to *p*-values, Chi^2^ tests were conducted.

**Table 2:** Correlates of loneliness, subjective isolation, and objective social isolation. Results of multiple linear regressions.

Independent Variables	Loneliness	Perceived Social Isolation	Objective Social Isolation
Age (in years)	−0.00	−0.02 +	0.01
	(0.01)	(0.01)	(0.07)
Family situation:—Living together: Married or in partnership (Reference category: Other including [living separately: married or in partnership; divorced; single; widowed])	−0.30 *	0.04	−0.63
	(0.14)	(0.16)	(1.21)
School education:—General or subject-specific university entrance qualification (e.g., “Abitur”) (Reference: Lower school education including [Completion of polytechnic secondary school; Currently in school education; Secondary school diploma; Intermediate school leaving certificate (e.g., “Realschulabschluss”); Without general school leaving certificate])	−0.47 **	−0.45 *	2.67 *
	(0.15)	(0.18)	(1.21)
Religious affiliation: —Having a religious affiliation including [Buddhism; Christianity; Islam; Other] (Reference category: Non-denominational)	−0.17	−0.07	0.36
	(0.15)	(0.18)	(1.18)
Labor force participation:—Full-time employed (Reference category: Unemployed)	−0.14	−0.75 ***	−0.20
	(0.17)	(0.22)	(1.72)
— Other including [Part-time employed; Marginally employed (450-euro job or mini-job); Retired/early retirement; Other; In retraining; In vocational training/apprenticeship]	−0.20	−0.56 **	0.74
	(0.17)	(0.21)	(1.48)
Already having a gender reassignment surgery:—Yes (Reference category: No)	0.30 *	0.06	−1.69
	(0.15)	(0.17)	(1.10)
Frequency of sports activities:—Less than one hour a week (Reference category: No sports activity)	−0.07	0.00	1.05
	(0.20)	(0.24)	(1.54)
—Regularly, 1–2 h a week	0.04	0.45 *	0.79
	(0.17)	(0.23)	(1.64)
—Regularly, 3–4 h a week	−0.02	0.04	1.05
	(0.20)	(0.22)	(1.46)
—Regularly, more than 4 h a week	0.36 *	0.28	0.15
	(0.18)	(0.26)	(1.73)
Migration background: —Yes (Reference category: No)	0.16	0.06	−0.33
	(0.22)	(0.26)	(1.50)
Self-rated health (from 1 = very bad to 5 = very good)	−0.18 *	−0.15	1.03
	(0.08)	(0.12)	(0.73)
Having at least one chronic disease: —Yes (Reference category: No)	0.11	0.31 +	−3.73 **
	(0.17)	(0.19)	(1.30)
Constant	3.50 ***	3.97 ***	10.98 ***
	(0.42)	(0.63)	(3.21)
R^2^	0.34	0.35	0.30
Observations	104	104	104

Notes: *** *p* < 0.001, ** *p* < 0.01, * *p* < 0.05, + *p* < 0.10; With regard to addressing missing data, FIML was used. Unstandardized beta-coefficients are displayed; robust standard errors are in parentheses.

## Data Availability

The datasets analyzed during the current study are not publicly available due to ethical restrictions involving patient data but are available from the corresponding author upon reasonable request.
